# Data from an integrative approach decipher the surface proteome *of Propionibacterium freudenreichii*

**DOI:** 10.1016/j.dib.2014.08.009

**Published:** 2014-09-21

**Authors:** Caroline Le Maréchal, Vincent Peton, Coline Plé, Christophe Vroland, Julien Jardin, Valérie Briard-Bion, Gaël Durant, Victoria Chuat, Valentin Loux, Benoit Foligné, Stéphanie-Marie Deutsch, Hélène Falentin, Gwénaël Jan

**Affiliations:** a INRA, UMR1253 STLO, Science et Technologie du Lait et de l׳Œuf, F-35042 Rennes, France; b GROCAMPUS OUEST, UMR1253 STLO, F-35042 Rennes, France; c Lactic acid Bacteria & Mucosal Immunity, Center for Infection and Immunity of Lille, Institut Pasteur de Lille, U 1019, UMR 8204 Université Lille Nord de France, 1 rue du Pr Calmette, BP 245, F-59019 Lille, France; d INRA, UMR1253 STLO, CIRM-BIA, F-35042 Rennes, France; e INRA, UR MIG, F-78352 Jouy-en-Josas, France

## Abstract

The surface proteins of the probiotic *Propionibacterium freudenreichii* were inventoried by an integrative approach that combines *in silico* protein localization prediction, surface protein extraction, shaving and fluorescent CyDye labeling. Proteins that were extracted and/or shaved and/or labeled were identified by nano-LC–MS/MS following trypsinolysis. This method’s combination allowed to confirm detection of true surface proteins involved in host/probiotic interactions. The data, supplied in this article, are related to the research article entitled “Surface proteins of *P. freudenreichii* are involved in its anti-inflammatory properties” (Le Maréchal et al., 2014 [6]).

**Table:** **Specifications Table**.

Subject area	*Microbiology, Food Technology*
More specific subject area	*Probiotics, fermented foods*
Type of data	*Tables and figures*
How data was acquired	*2-D PAGE: Multiphor II and Ettan DALTtwelve* (*GE Healthcare*)
	*Image analysis: Image-Master 2D*
	*Mass spectrometry: QSTAR XL* (*Applied Biosystem*)
Data format	*Analyzed*
Experimental factors	*Dairy propionibacteria were grown in milk aqueous phase and directly subjected to guanidine extraction, trypsin shaving or CyDye labeling* (*see*Fig. 1)
Experimental features	*All the samples were analyzed by nano-LC coupled to MS/MS*
Data source location	*City, Country and/or Latitude & Longitude* (*& GPS coordinates*) *for collected samples/data if applicable*
Data accessibility	*Within this article*

**Table:** **Value of the data**.

• Probiotic bacteria are analyzed after growth in milk aqueous phase, not in laboratory medium as it is usually done. • A combination of 3 proteomic methods is used to validate surface localization of the identified proteins. • The data point out candidate genes for functional genomics. • The data open new perspectives to identify molecular mechanisms of probiotic/host interactions.

## 1 Data, experimental design, materials and methods [6]

### 1.1 Data

**Fig. 1:**
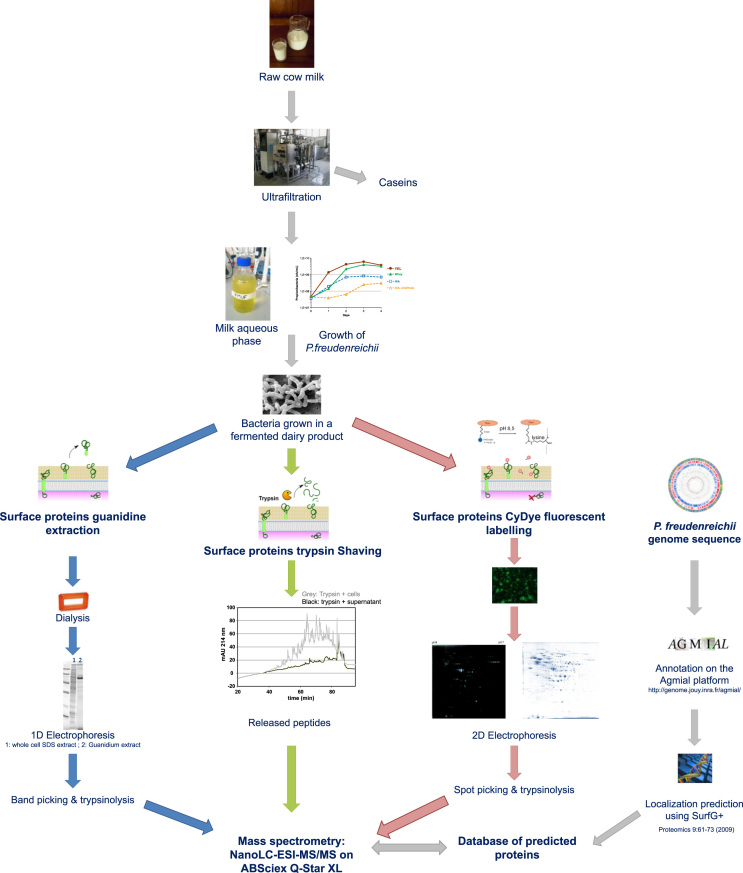
Experimental flowchart of the surface protein analysis procedure.

Fig. 1 summarizes the 3 proteomic methods used to acquire data on the surface proteins of the probiotic *Propionibacterium freudenreichii*. Data acquired by guanidine hydrochloride extraction are presented in Supplementary Table 1. Data acquired by enzymatic shaving using trypsin are presented in Supplementary Table 2. Data acquired by in situ fluorescent labeling usingCyDye DIGE Fluor Cy5 minimal Dye are presented in Supplementary Table 3.

### 1.2 Genome sequencing, annotation and bio-informatics

The genome of *P. freudenreichii* ITG P20 was previously sequenced and annotated [4] and the draft assembly deposited in the European Nucleotide Archive (EMBL-EBI accession number: CONTIGS: CCBE010000001–CCBE010000111; SCAFFOLDS:HG975453–HG975511). Prediction of subcellular localization of encoded proteins were done in this work using SurfG+ [2].

### 1.3 Whole-cell protein extracts

Whole cell SDS extracts were prepared by disruption of cells in SDS according to a procedure modified from one previously described [7].

### 1.4 Extraction of surface proteins non-covalently bound to the cell wall using guanidine hydrochloride

Surface layer proteins were extracted according to a procedure modified from one previously described [8]. 100 mL of stationary phase culture (see above) were harvested by centrifugation (6000*g*, 10 min, 4 °C) and washed in an equal volume of PBS prior to resuspension in 5 M guanidine hydrochloride to a final OD_650_ of 20. The suspension was incubated 15 min at 50 °C prior to centrifugation (21,000*g*, 20 min, 30 °C) to eliminate cells. The supernatant was then dialyzed exhaustively against 0.1% SDS in distilled water during 24 h at 4 °C using 10,000 kDa cutoff Slide-A-Lyer^®^ Dialysis Cassette (ThermoScientific, Rockford, USA) prior to proteomic investigations.

### 1.5 Enzymatic shaving of surface proteins

100 mL of stationary phase culture (see above) were harvested by centrifugation (6000*g*, 10 min, 4 °C) and washed in an equal volume of PBS [pH 8.5] containing 5 mM DTT prior to resuspension in 1/10 volume of the same buffer. Sequencing grade modified trypsin (V5111, Promega, Madison, USA) was dissolved in the same buffer (qsp 0.2 g/L) and added to the bacterial suspension. “Shaving” was performed for 1 h at 37 °C in a 0.5 mL reaction volume containing 5×10^9^ bacteria and 4 μg of trypsin, with gentle agitation (180 rpm). Bacteria were removed by centrifugation (8000*g*, 10 min, 20 °C) and the supernatant filtered (0.2 µm, Nalgene) prior to addition of 1 μg of trypsin to complete digestion of released peptides (16 h, 37 °C). Trypsin digestion of the supernatant was stopped by adding trifluoroacetic acid to a final concentration of 0.15% (v/v). The supernatants containing peptides were then concentrated in a Speed-Vac concentrator prior to nano-LC–MS/MS analysis.

### 1.6 Nano-LC–MS/MS analyses

For trypsinolyzed proteins, nano-LC experiments were performed using an on-line liquid chromatography tandem mass spectrometry (MS/MS) setup using a Dionex U3000-RSLC nano-LC system fitted to a QSTAR XL (MDS SCIEX, Ontario, Canada) equipped with a nano-electrospray ion source (ESI) (Proxeon Biosystems A/S, Odense, Denmark). Samples were first concentrated on a PepMap 100 reverse-phase column (C18, 5 μm particle size, 300-μm inner diameter (i.d.) by 5 mm length) (Dionex, Amsterdam, The Netherlands). Peptides were separated on a reverse-phase PepMap 100 column (C18, 3 μm particle size, 75 μm i.d. by 150 mm length) (Dionex) at 35 °C, using solvent A (2% (vol/vol) acetonitrile, 0.08% (vol/vol) formic acid, and 0.01% (vol/vol) TFA in deionized water) and solvent B (95% (vol/vol) acetonitrile, 0.08% (vol/vol) formic acid, and 0.01% (vol/vol) TFA in deionized water). A linear gradient from 10% to 50% of solvent B in 40 min was applied for the elution at a flow rate of 0.3 μL/min. Eluted peptides were directly electrosprayed into the mass spectrometer operated in positive mode. A full continuous MS scan was carried out followed by three data-dependent MS/MS scans. Spectra were collected in the selected mass range 400–2000*m*/*z* for MS and 60–2000 *m*/*z* for MS/MS spectra. The three most intense ions from the MS scan were selected individually for collision-induced dissociation (1+ to 4+ charged ions were considered for the MS/MS analysis). The mass spectrometer was operated in data-dependent mode automatically switching between MS and MS/MS acquisition using Analyst QS 1.1 software. The instrument was calibrated by multipoint calibration using fragment ions that resulted from the collision-induced decomposition of a peptide from β-casein, β-CN (193–209). The proteins present in the samples were identified from MS and MS/MS data by using MASCOT v.2.2 software for search into two concatenated databases: (i) a homemade database containing all the predicted proteins of the *P. freudenreichii* strain CIRM-BIA 129 used in this study and (ii) a portion of the UniProtKB database corresponding to *P. freudenreichii*. Search parameters were set as follows. A trypsin enzyme cleavage was used, the peptide mass tolerance was set to 0.2 Da for both MS and MS/MS spectra, and two variable modifications (oxidation of methionine and deamidation of asparagine and glutamine residues) were selected. For each protein identified in NanoLC-ESI-MS/MS, a minimum of two peptides with MASCOT score corresponding to a *P* value below 0.05 were necessary for validation with a high degree of confidence. For automatic validation of the peptides from MASCOT search results, the 1.19.2 version of the IRMa software was used [3].

### 1.7 In situ surface labeling

The surface labeling procedure was adapted from Hagner-McWhirter et al. [5]. Bacteria were grown and harvested as described above and washed in an equal volume of ice-cold PBS containing 33 mM Tris–HCl [pH 8.5] prior to centrifugation. Bacteria were resuspended in 1/10 volume of ice-cold labeling buffer (PBS containing, 1 M urea, 33 mM Tris–HCl [pH 8.5]). Labeling was performed on ice, in the dark, in a 1 mL reaction volume containing 10^10^ live and intact bacteria, and 200 pmol of CyDye DIGE Fluor Cy5 minimal dye (GE Healthcare, Orsay, France). Labeling was stopped by adding 1 μmol of Lysine to quench the dye. Labeled bacteria were centrifuged and washed in PBS (pH 7.4), centrifuged and resuspended in SDS lysis buffer (50 mM Tris–HCl [pH 7.5], 0.3% SDS, 200 mM DTT) prior to whole cell protein extraction as described above.

### 1.8 Two-dimensional imaging and spot picking

Whole-cell protein SDS extracts of labeled bacteria were precipitated using the 2D Clean-Up Kit (GE Healthcare) prior to dissolution in destreak rehydration solution (100 μl per sample) added with 2% (w/v) ampholyte containing buffer (IPG-Buffer 4–7, GE Healthcare). Isoelectric focusing was carried out using pH 4–7, 18 cm, Immobiline Dry Strips on a Multiphor II electrophoresis system (GE Healthcare) for a total of 60 kV h using a standard procedure described previously [1]. The second dimensional separation was performed on the Ettan™ DALTtwelve electrophoresis system (GE Healthcare) using 14% acrylamide separating gels without a stacking gel at a voltage of 50 V for 1 h and 180 V for about 7 h. Fluorescent images of the gels were immediately acquired on a Typhoon PhosphorImager (GE Healthcare) using the appropriate laser excitation for Cy5 fluorescence. Gels were then fixed and Coomassie Blue-stained as described above. Visible images were acquired on an ImageScanner III (GE Healthcare). Images were further analyzed using Image- Master 2D software. Fluorescent profiles of 2-DE-separated proteins were reproducible in at least three individual experiments. Fluorescent and Coomassie-Blue visible images of the 2D electrophoresis gels were matched to detect surface-exposed proteins. Fluorescent spots corresponding to surface-exposed proteins were excised from 2-DE gels as previously described [7] when detectable by Coomassie-Blue staining. Proteins were identified by tandem mass spectrometry (MS/MS) after an in-gel trypsin digestion adapted from Shevchenko [9]. Briefly, gel pieces were washed with acetonitrile and ammonium bicarbonate solution, and then dried under vacuum in a SpeedVac concentrator (SVC100H-200; Savant, Thermo Fisher Scientific, Waltham, MA, USA). In-gel trypsin digestion was performed overnight at 37 °C and stopped with spectrophotometric-grade trifluoroacetic acid (TFA) (Sigma-Aldrich). The supernatants containing peptides were then vacuum dried in a Speed-Vac concentrator and stored at −20 °C until mass spectrometry analysis. Nano-LC–MS/MS analysis was as described above.
