# Supplementary material for: Data from an integrative approach decipher the surface proteome of Propionibacterium freudenreichii
Source: Data Brief. 2014 Sep 21;1:46–50. doi: 10.1016/j.dib.2014.08.009 (PMC4459863; doi:10.1016/j.dib.2014.08.009)
Supplement: Supplementary file 1 — Supplementary data [file mmc1.zip › SuppTable-1.Jan.docx]

**Table 1.** Proteins identified after Guanidine Hydrochloride extraction

| **Locus Tag** | **Description** | **Gene** | **Function** | **Molecular Weight (kDa)^(a)^** | **SurfG+ predicted localisation** | **Mascot Score****^(b)^** | **Number of unique peptides** | **Coverage (%)^(c)^** |
| --- | --- | --- | --- | --- | --- | --- | --- | --- |
| PFCIRM129_12235 | Internalin A | inlA | Miscellaneous | 145,5 | PSE | 3006,8 | 34 | 36,8 |
| PFCIRM129_05460 | Surface protein with SLH domain | slpE | Cell wall | 59,2 | PSE | 442,0 | 6 | 16,0 |
| PFCIRM129_09350 | Surface layer protein A | slpA | Cell wall | 58,3 | PSE | 254,2 | 3 | 9,7 |
| PFCIRM129_00700 | Surface layer protein B | slpB | Cell wall | 56,8 | PSE | 880,2 | 10 | 33,3 |
| PFCIRM129_11445 | Large surface protein A | lspA | Cell wall | 96,1 | SECRETED | 260,7 | 3 | 5,8 |

(a) Proteins molecular weights were automatically predicted from the corresponding genes on the Agmial annotation platform

(b) Mascot software calculates the score of a protein as the sum of the score of each identified peptides for this protein. The score of a peptide is calculated as -10*LOG10(P), where P is the probability of the match to be a random event

(c) Coverage of a protein is calculated as the percentage of the amino acid sequence included in the peptides identified
