# Supplementary material for: Data from an integrative approach decipher the surface proteome of Propionibacterium freudenreichii
Source: Data Brief. 2014 Sep 21;1:46–50. doi: 10.1016/j.dib.2014.08.009 (PMC4459863; doi:10.1016/j.dib.2014.08.009)
Supplement: Supplementary file 1 — Supplementary data [file mmc1.zip › SuppTable-2.Jan.docx]

**Table 2.** Proteins identified after enzymatic shaving with trypsin

| **Locus Tag** | **Description** | **Gene** | **Function** | **Molecular Weight (kDa) ^(a)^** | **SurfG+ predicted localisation** | **Mascot Score^(b)^** | **Number of unique peptides** | **Coverage (%)^(c)^** |
| --- | --- | --- | --- | --- | --- | --- | --- | --- |
| PFCIRM129_12235 | Internalin A | inlA | Miscellaneous | 145,5 | PSE | 3614,2 | 37 | 54,0 |
| PFCIRM129_05460 | Surface protein with SLH domain | slpE | Cell wall | 59,2 | PSE | 1180,0 | 14 | 45,6 |
| PFCIRM129_09350 | Surface layer protein A | slpA | Cell wall | 58,3 | PSE | 173,4 | 3 | 7,7 |
| PFCIRM129_00700 | Surface layer protein B | slpB | Cell wall | 56,8 | PSE | 1128,2 | 12 | 48,0 |
| PFCIRM129_11445 | Large surface protein A | lspA | Cell wall | 96,1 | SECRETED | 1310,5 | 13 | 36,4 |
| PFCIRM129_11920 | Secreted transglycosydase |  | Cell wall | 20,1 | PSE | 190,1 | 2 | 39,1 |
| PFCIRM129_10570 | Penicillin-binding protein | ponA | Cell wall | 77,9 | SECRETED | 1040,7 | 12 | 37,4 |
| PFCIRM129_09980 | Peptidyl-prolyl cis-trans isomerase | prsA | Protein folding | 35,9 | SECRETED | 240,3 | 3 | 15,3 |
| PFCIRM129_09060 | Hypothetical secreted protein |  | Protein of unknown function | 26,2 | PSE | 125,5 | 2 | 13,7 |
| PFCIRM129_08670 | Cell-wall peptidase |  | Cell wall | 58,7 | SECRETED | 783,7 | 9 | 25,7 |
| PFCIRM129_08120 | Solute binding protein of the ABC transport system | bopA | Transport/binding of proteins/peptides | 61,4 | PSE | 847,0 | 10 | 27,8 |
| PFCIRM129_08025 | Resuscitation-promoting factor | RpfB | Adaptation to atypical conditions | 37,7 | SECRETED | 770,0 | 8 | 37,7 |
| PFCIRM129_05625 | Binding protein of iron ABC transporter | fepC2 | Transport/binding of inorganic ions | 36,1 | SECRETED | 177,2 | 2 | 8,5 |
| PFCIRM129_08275 | Elongation factor Tu | tuf | Translation elongation | 43,6 | CYTOPLASMIC | 192,2 | 2 | 7,8 |
| PFCIRM129_11455 | Hypothetical protein |  | Protein of unknown function | 36,4 | CYTOPLASMIC | 150,2 | 2 | 8,7 |
| PFCIRM129_07835 | 60 kDa chaperonin 1 | groL1 | Protein folding | 56,1 | CYTOPLASMIC | 132,6 | 2 | 8,1 |

(a) Proteins molecular weights were automatically predicted from the corresponding genes on the Agmial annotation platform

(b) Mascot software calculates the score of a protein as the sum of the score of each identified peptides for this protein. The score of a peptide is calculated as -10*LOG10(P), where P is the probability of the match to be a random event

(c) Coverage of a protein is calculated as the percentage of the amino acid sequence included in the peptides identified
