# Supplementary material for: Data from an integrative approach decipher the surface proteome of Propionibacterium freudenreichii
Source: Data Brief. 2014 Sep 21;1:46–50. doi: 10.1016/j.dib.2014.08.009 (PMC4459863; doi:10.1016/j.dib.2014.08.009)
Supplement: Supplementary file 1 — Supplementary data [file mmc1.zip › SuppTable-3.Jan.docx]

**Table 3.** Proteins identified after *in situ* fluorescence labelling using CyDye DIGE Fluor Cy5 minimal dye

| **Locus Tag** | **Description** | **Gene** | **Function** | **Molecular Weight (kDa) ^(a)^** | **SurfG+ predicted localisation** | **Mascot Score^(b)^** | **Number of unique peptides** | **Coverage (%)^(C)^** | **Spot Number^(d)^** |
| --- | --- | --- | --- | --- | --- | --- | --- | --- | --- |
| PFCIRM129_12235 | Internalin A | inlA | Miscellaneous | 145,5 | PSE | 79,4 | 2 | 3,7 | 6 |
| PFCIRM129_05460 | Surface protein with SLH domain | slpE | Cell wall | 59,2 | PSE | 129,0 | 2 | 4,5 | 4 |
| PFCIRM129_09350 | Surface layer protein A | slpA | Cell wall | 58,3 | PSE | 523,6 | 9 | 26,4 | 7 |
| PFCIRM129_00700 | Surface layer protein B | slpB | Cell wall | 56,8 | PSE | 721,9 | 10 | 48,5 | 7 |
| PFCIRM129_08275 | Elongation factor Tu | tuf | Translation elongation | 43,6 | CYTOPLASMIC | 527,1 | 7 | 25,3 | 9 |
| PFCIRM129_11455 | Hypothetical protein |  | Protein of unknown function | 36,4 | CYTOPLASMIC | 192,3 | 2 | 20,0 | 12 |
| PFCIRM129_07835 | 60 kDa chaperonin 1 | groL1 | Protein folding | 56,1 | CYTOPLASMIC | 299,6 | 5 | 9,6 | 4 |
| PFCIRM129_03120 | Heat shock protein 20 2 | hsp20 2 | Protein folding | 16,8 | CYTOPLASMIC | 380,5 | 6 | 11,9 | 19 |
| PFCIRM129_08280 | Elongation factor G (EF-G) | fusA | Translation elongation | 76,5 | CYTOPLASMIC | 277,1 | 5 | 5,0 | 6 |
| PFCIRM129_07955 | GTP phosphohydrolase |  | Translation elongation | 75,1 | CYTOPLASMIC | 440,2 | 7 | 20,0 | 6 |
| PFCIRM129_07645 | Malate dehydrogenase | Mdh | Metabolism of carbohydrates and related molecules | 34,8 | CYTOPLASMIC | 782,4 | 11 | 28,2 | 141 |
| PFCIRM129_11225 | FeS assembly protein SufB | sufB | Transport/binding proteins and lipoproteins | 53,8 | CYTOPLASMIC | 521,4 | 9 | 15,6 | 5 |
| PFCIRM129_07235 | Methylmalonyl-CoA mutase small subunit | mutA | Specific carbohydrate metabolic pathway | 69,5 | CYTOPLASMIC | 307,5 | 5 | 15,5 | 3 |
| PFCIRM129_01500 | Pyruvate phosphate dikinase | Ppdk | Metabolism of carbohydrates and related molecules | 95,7 | CYTOPLASMIC | 121,3 | 2 | 6,9 | 1 |
| PFCIRM129_05475 | DNA polymerase III, beta chain | dnaN | DNA replication | 41,4 | CYTOPLASMIC | 169,0 | 3 | 15,9 | 10 |
| PFCIRM129_04980 | D-alanine--D-alanine ligase | ddlA | Cell wall | 40,4 | CYTOPLASMIC | 155,0 | 2 | 14,3 | 13 |
| PFCIRM129_11075 | Elongation factor Ts (EF-Ts) | tsf | Translation elongation | 28,8 | CYTOPLASMIC | 285,1 | 5 | 31,7 | 16 |
| PFCIRM129_00390 | Cysteine synthase 2 | cys2 | Metabolism of amino acids and related molecules | 33,5 | CYTOPLASMIC | 511,8 | 8 | 30,1 | 15 |
| PFCIRM129_11210 | ABC-type transport system | sufC | Transport/binding proteins and lipoproteins | 26,8 | CYTOPLASMIC | 89,2 | 2 | 10,7 | 18 |
| PFCIRM129_01960 | Inositol-1-phosphate synthase |  | Specific carbohydrate metabolic pathway | 39,1 | CYTOPLASMIC | 141,4 | 2 | 6,0 | 11 |
| PFCIRM129_04355 | Translation factor SUA5 |  | Translation initiation | 22,6 | CYTOPLASMIC | 79,4 | 2 | 3,7 | 17 |
| PFCIRM129_03920 | Pyridine nucleotide-disulphide Oxidoreductase | merA | Metabolism of coenzymes and prosthetic groups | 48,1 | CYTOPLASMIC | 129,0 | 2 | 4,5 | 8 |
|  |  |  |  |  |  |  |  |  |  |

(a) Proteins molecular weights were automatically predicted from the corresponding genes on the Agmial annotation platform

(b) Mascot software calculates the score of a protein as the sum of the score of each identified peptides for this protein. The score of a peptide is calculated as -10*LOG10(P), where P is the probability of the match to be a random event

(c) Coverage of a protein is calculated as the percentage of the amino acid sequence included in the peptides identified

(d) These numbers refer to the spots labeled on figures 3D and 3E
